# Atlanta Academy of Medicine

**Published:** 1872-11

**Authors:** Jas. B. Baird

**Affiliations:** Reporter


### ATLANTA ACADEMY OF MEDICINE.

JAS. B. BAIRD, M.D., REPORTER.

[Extract from Minutes, Nov. 11th, 1872—Dr. J. P. Logan, President, in the Chair.]

Dr. A. W. Griggs, of West Point, being present, reported a fever of an unusual type prevailing sporadically in the city of West Point, and surrounding country. The attack is ushered in by premonitory symptoms—first by a rigor, but no decided chill; active from the first; pulse full and strong, and uniformly about ninety-six per minute; tongue flat, rounded end, not swollen, covered with a white fur, and in a few cases it presents a reddish glow; papulæ distinct. The fever is remittent. Bowels constipated at first; pain in the head; wry neck. In three to five days, epistaxis occurs, which relieves the pain in the head; the eyes are injected—lids œdematous; alvine evacuations are liquid; urine high-colored, and acid. Epistaxis is a prominent symptom, and in one case, it occurred regularly every day at five o'clock in the afternoon. The fever was unyielding. Mercury and quinine seemed to have little effect; the latter, given to the amount of twenty or twenty-five grains during the apyrexia, would keep the fever off while the patient was under its influence. These doses did not increase the headache, Epigastric tenderness slight; no tympanitis; no paralysis of the tongue. Improvement generally commenced on the twelfth or fifteenfh day; convalescence usually declared by the eighteenth day. Anorexia is prominent throughout.

But one fatal case has occurred—a girl aged thirteen years; dyspeptic. Was attacked Sunday with epistaxis, which continued so long that gallic acid, snuffed up the nostril, was prescribed, and twenty trops of diluted elixir vitriol, with one grain of quinine, administered every hour. The hemorrhage was relieved for the time, but recurred on Monday. In his absence, Dr. Todd was sent for, and it became necessary to plug the nares. Hæmaturia was then developed. On his return he was called, and found, upon examination, large purpuric spots, the size of his hand, on her body; some were red, and smaller. Nitrate of iron and ergot were prescribed. The nitrate was soon substituted by the muriated tincture of iron, and after a few doses, the ergot was discontinued. The iron was first given in ten, afterward fifteen and thirty drops, every three hours. The urine gradually became clear. On Friday morning, she seemed to be doing well; took nourishment. The iron was administered at longer intervals, but she died at four o'clock P.M. that day.

In only one case was there gastric irritability, and that one was complicated with bronchitis. In one case, neuralgia of the face preceded the development of the fever; in another, there was passive hemorrhage from the bowels. Intermittent fever prevailed in that section not more extensively than usual, and he called this pernicious remittent fever, on account of its obstinacy.

Dr. John G. Westmoreland's idea of pernicious malarial fever was, that it was a state of great prostration and violent congestion, usually resulting in death in two or three days, generally during the second paroxysm. The fever referred to by Dr. Griggs, he thought, was the same that prevailed so extensively in 1847. Delirium was sometimes present. It was thought at first to be typhoid fever. Its duration was about two weeks. Ordinary treatment seemed to be futile.

Dr. Griggs remarked that the fever alluded to by Dr. Westmoreland prevails in West Point, and is known as congestive or pernicious intermittent fever. It generally proves fatal in the second paroxysm—frequently during the first. He would like to hear the opinion of the Academy on the case reported.

Dr. Westmoreland observed that it was not a rare case. The patient was anæmic; her blood was poor—this favors heart clot—or in raising herself in bed, may have had an attack of syncope, from which she did not recover. Has seen many cases of the fever described by Dr. Griggs. Objected to the name used by the Doctor. It is the same disease called by Dr. Mitchell, of Philadelphia, solar fever. Although it is malarial, quinine does not control it. Thinks the brain is more affected than in ordinary malarial fevers. Has seen cases this fall which caused him at first to suspect typhoid fever. It disappeared under the use of quinine, but reappeared when its administration was suspended.

Dr. Griggs thought quinine did not exert its specific influence in an inflammatory state of the system.

Dr. Love inquired if there had been a recurrence or relapse in this fever, or had a sufficient time elapsed to determine?

Dr. Griggs replied that several weeks had elapsed since recovery, in some cases, and no relapse had taken place.

Dr. W. F. Westmoreland reported a case of uterine hemorrhage. A lady, after a violent attack of fever, miscarried, giving birth to a seven months' child. The labor pains were possibly produced by large doses of quinine. The labor lasted for two or three hours. A few minutes before delivery took place, he made an examination, and, from the condition of the os uteri, expressed the opinion that it would be several hours before its completion. The next pain expelled the child. Immediately after severing the cord, as is his custom, he proceeded to remove the placenta. An alarming hemorrhage simultaneously occurred. In two minutes, she was almost pulseless. The blood welled up to a frightful amount. Clots were removed, and yet the flow continued. He grasped the uterus in his hand, but it refused to contract. He then seized a piece of ice about the size of a hen's egg, and thrust it into the uterus. This produced contraction and an immediate cessation of the hemorrhage. When the first melted, another piece was introduced, and the operation continued for half an hour. The hemorrhage did not recur. He stated that the President was familiar with the case of fever preceding the hemorrhage, and requested a report of the attack from him.

To this request, Dr. Logan replied by saying that the lady had an attack of fever of an obscure type, from which she was convalescent, being able to sit up for a day or two, when she relapsed, the symptoms being more violent than during the first attack. Chills were distinct, but irregular; no typhoid symptoms; pulse full—ninety-six per minute; vomiting almost constant. During one of these exacerbations, delivery occurred. Near the residence is a large sewer, having three openings within a few hundred feet of her dwelling. The lot itself is low. He believes the fever was caused by emanations from this sewer. Several members of the family were affected in the same way. Thinks it strongly resembles "relapsing fever." Dr. Westmoreland calls it *sewer* fever.

Dr. Connally had, in two instances, checked alarming uterine hemorrhage by the introduction of ice into the uterus. He believes the lives of his patients were saved by it.

The case of supposed cerebro-spinal meningitis, reported by him two weeks ago, did well up to the last twenty-four hours; since that time has retained urine, and has lost ground rapidly. Thinks he will die within forty-eight hours.

Dr. John G. Westmoreland desired to take advantage of the occasion to insist upon active treatment in meningitis. Let alone opium and quinine; results from this treatment have been unfavorable. Advises the most active antiphlogistic treatment. As soon as symptoms of meningitis appear, bleed from the arm, and bleed freely. If the patient has no pulse, bleed to give him one. For those cases which do not react, he has no treatment.

Dr. Griggs never lost a case in which he used the lancet, and never saved a case in which he did not. At the same time, a too early resort to blood-letting is injudicious. Bleeds when opisthotonos appears. Photophobia and hyperæsthesia of one side have been prominent symptoms in the cases which have fallen under his observation. In one case, there was paralysis of the right arm and left leg. He referred to the remarkably rapid and firm coagulation of blood drawn from cases suffering from meningitis.

---

November 18, 1872.

Dr. E. L. Connally reported the following history of a case of cerebro-spinal miningitis:

Was called on the 25th of October to see Henry Mayson, colored, 22 years old; well developed; by occupation a drayman. Had slight attack of fever in early summer; has been in feeble health since; has been sick for a week or more; suffered a good deal from "crick in neck." Two days before I saw him, in attempting to rise from the bed he fell on the floor and was unable to get back in the bed without help. Found him lying quietly on the bed and unable to move, even to turn himself in bed. Muscular system of upper and lower extremities completely paralysed, except slight motion in fore arms and fingers; sensation normal; pain in head; stiffness of the neck, extending into the back; no opisthotonos; slight pain in breast and arms; occasional paroxysms of hiccough and vomiting; pulse 68 per minute—normal; respiration normal; cerebral functions perfect; pupils normal; bowels constipated; tongue loaded with thick, creamy coat; appetite good; urine not examined.

Ordered: ℞.—Calomel, grs. xxv.

Morphia, gr. $\frac{1}{3}$.

S.—Make one powder, to be taken at once. Blister to back of neck.

*Twenty-sixth.*—Rested well during night; pulse 55, morning and evening; rather soft than resisting; free from pain; takes food well, but it soon induces hiccough, and vomiting follows; can eat again, however, in a few moments.

Ordered: ℞.—Ol. ricini, ℥ j.

*Twenty-seventh.*—Pulse 55; has better use of head and neck, paralysis of the muscles increased; cannot even extend the fingers; free from pain; hiccough troublesome.

Ordered: ℞.—Calomel, grs. x.
Pulv. Opium, grs. v.

S.—Make twenty powders; one to be taken every three hours.

*Twenty-eighth.*—Pulse 48, morning and evening; rather soft than resisting, but would be considered a normal pulse in every respect, rate excepted; respiration 14; cerebral functions perfect; temperature not taken. Ordered three grains quinia every four hours; blister to be applied to back of the neck, to extend over dorsal vertebræ.

*Twenty-ninth.*—Pulse 58; otherwise unchanged; evacuates bowels and bladder without any difficulty.

*November Fifth.*—Pulse 80; with less volume; hiccough and vomiting subsiding; bowels constipated; otherwise but little change. Ordered: Ol. ricini, ℥ j, which had to be repeated.

*November Eighth.*—Pulse 100; growing weaker; ordered stimulants, and blister re-applied to back of neck, to extend over the dorsal vertebræ.

*November Eleventh.*—Failed to evacuate his bladder; performed catheterism; deglutition difficult; mental faculties perfect; pulse 125. Died at 9 P.M.

*Post-mortem Eighteen Hours After Death.*—Body not much emaciated; abdomen very much swollen; excessive discharge from mouth and nose of dark serous fluid. On removing the skull cap, there was undue congestion of the meninges, particularly the posterior part; the substance of the brain was also congested; lateral ventricles contained half ounce turbid serum. There was considerable exudation of lymph and serum at the base of the brain; intense congestion of the meninges, covering the spinal cord and medulla oblongata, with extensive fibrilated attachment of the cord. There was also an exudation of gelatinous substance on the cord. Thoracic and abdominal viscera not opened.

I am indebted to Drs. W. F. Westmoreland, A. W. Griggs and Ch. Raushenberg, for their presence and assistance during the post-mortem examination.

I have no doubt in my mind that this was a case, primarily, of spinal meningitis, subsequently extending itself.

There are some points of interest presented: The pathological phenomenon of complete paralysis of the muscles of the extremities, and at the same time sensation remaining perfect in them. Pathologists account for this, I believe, in the exemption from disease of the roots of the nerves; and again, may not incipient meningitis or meningitis proper exist while in apparent health? For, in this case but little complaint was made; indeed, he never took his bed until after he was paralyzed.

The "crick" was in his neck a week before he was paralyzed. This was a premonitory symptom in this case, and a congestion of the meninges of the cord no doubt existed at that time. Now, the question arises, whether or not there was an effusion at the time he was paralyzed, or was it produced by the increased congestion of the meninges, and the effusion subsequently taking place? This seems to me to be important in the treatment of such cases.

Dr. John G. Westmoreland did not think that effusion was necessary to account for the paralysis in this case. He thought that it was produced by congestion—engorgement of the meninges of the cord.

Dr. W. F. Westmoreland.—There is no doubt but that this man had so-called cerebro-spinal meningitis. The system is impressed with the peculiar poison, and it may exhibit itself as pleurodynia, pleuritis, sore throat, etc. It may at any time attack the cord, but thinks that an active inflammatory condition cannot exist without symptoms proportionate to its severity.

As an example: we may, upon exposure, contract a sore throat in five minutes, and suffer severe pain. Thinks there is the same kind of determination of the blood to the meninges in the so-called cerebro-spinal meningitis. Thinks that paralysis in this case was not dependent upon effusion, but congestion. There was not time enough after violent symptoms appeared for effusion to take place.

Dr. John G. Westmoreland replied, that even if the same general cause should produce meningitis, pleurodynia, pleuritis, bronchitis, etc., they should not all be called meningitis. They are different diseases.

Dr. Rauschenberg remarked, that the state of things which he had before him in the limited number of cases of cerebro-spinal meningitis which came to his observation so far, did not leave the impression upon his mind that this disease, or at least what he saw of it, represented a sthenic inflammation. He treated one case six weeks, which finally recovered, and the distinctly paroxysmal, mostly intermittent exacerbation of all the prominent pathological phenomena characterizing the disease so distinctly developed in this and other cases—the invariably soft, easy, compressible and frequently irregular and intermittent pulse, did not reveal to him that state of the nervous system which—as it does in sthenic inflammation—reacts vigorously at the instance of an irritating impression, producing increased force and frequency of the heart's action, and a more or less tense, full and frequent pulse, while the local hyperæmia, with increased afflux of blood and enlargement of the capillaries, terminates in morbid metamorphosis, stasis and its local consequences—but rather the impression of a disease where a morbid influence (such as we have in infectious diseases) has poisoned the blood, and produces irregular and morbid innervation. He could not agree with the opinion, expressed in the last meeting of the Academy, that venesection deserved a trial whenever the existence of the usual symptoms of cerebro or cerebro-spinal trouble, as they occur in this disease, was well established, because—if the condition of the pulse manifests, with any degree of reliance, the state of the nerve-centers and the heart—a soft, frequent pulse, or a slow, otherwise normal, or slow, soft pulse, could never indicate that course of treatment. When blood is abstracted sometimes in peritonitis or other inflammations of the abdominal organs, and also in the commencement of pneumonia, when from extensive capillary engorgement, mechanical obstruction of the circulation and respiration, and diminished quantity of blood in the larger vessels, we meet with a small, frequent, but hard pulse, then we can bleed and lessen all these unfavorable qualities of the pulse. When we bleed in compression of the brain from mechanical injuries, where the pulse is slow but firm, we also improve the pulse; but when we bleed a patient with a soft, easy, compressible pulse, it will always get softer and the patient worse, because a soft pulse always indicates weakness, want of irritability, loss of power of the nervous system.

The anatomico-pathological changes, discovered by the post-mortem examinations of these cases, he did not consider an evidence of inflammation, in the common sense of the word. He could discover no good reason why local inflammatory action, local disturbance of nutrition, should invariably depend upon abnormally increased action of the vaso-motor and trophic nerves of the inflamed part, and upon active hyperæmia and an increase of the organic chemical process; and why diminished action of the same nerves, causing a loss of tone of the capillaries—consequently passive hyperæmia and an imperfect metamorphosis—could not as soon produce stasis, effusion and exudation. To this latter class of local nutritive disturbances, he counts those cases of epidemic cerebro-spinal meningitis which he has seen.

Dr. A. W. Griggs, of West Point, believes that it is agreed among pathologists that inflammation is perverted nutrition. Thinks that conclusive proof is afforded by the report just read, that it was a case of acute inflammation of the cerebro-spinal meninges. We have here congestion, effusion and lymph, clearly establishing the fact that inflammation existed. There were, undoubtedly, evidences of cerebral engorgement in its inception. Usually a chill ushers in the attack, and in his experience, the severity of the chill is an index of the gravity of the attack. Generally convulsions follow, sometimes there are none; then opisthotonos; an inflammatory condition of the conjunctiva is frequently observed; increased action of the carotid arteries. Pulse in the cases seen was always small—sometimes scarcely perceptible in the radial artery. True, there is perverted nervous action, but due, he thinks, to the condition of the blood. Diseased action is transferred to the nervous centers; it does not originate in them. He advises blisters to the spine, and calomel; but, as often repeated, the lancet is the most potent remedy of all. The remissions frequently observed are due to an inherent property of the nervous system. Blood-letting should not be practiced too soon, as he believes that, in the early stages of the disease, it favors effusion. The type presented by this disease may vary in different localities; sometimes no opportunity for using the lancet is offered, as these cases die in the very commencement of the attack. Thinks that in its inception, opium is admissible to abort—that is to prevent, not to cure inflammation, for it is not then established; in this stage, also, sinapisms to the spine. In one case, he found no pulse at the wrist. He bled from the temporal artery. The next day the pulse was so full that he bled again from the arm, and at the same time administered calomel; also veratrum viride to control vascular action, and quinine to correct intermittency.

Dr. A. K. Smith (United States Army) reported a case of acne rosacea in a man who did not drink, in which not only the follicles of the skin, but the connective tissue between them, was involved, and it was beginning to creep out on the alæ of the nose. The man had taken Fowler's solution for a long time, without benefit. A few weeks ago treatment with collodion was suggested while reading Niemeyer on mechanical treatment of certain diseases. He at once ordered the daily application of collodion to the parts affected. The nose began to grow whiter and whiter. The treatment has been persevered in for five weeks, and improvement continues.

Dr. W. F. Westmoreland hopes that Dr. Smith will report the progress of this case, for he has never seen any benefit from treatment, except a temporary improvement from the application of astringents.

